# The relationship between innate/adaptive immunity and gastrointestinal cancer : a multi-omics Mendelian randomization study

**DOI:** 10.1186/s12876-024-03284-x

**Published:** 2024-06-14

**Authors:** Chen-Xi Lv, Lin-Po Zhou, Ye-Bing Yang, Jing Shi, Fan-He Dong, Hao-Ran Wei, Yu-Qiang Shan

**Affiliations:** 1 The Fourth School of Clinical Medicine, Zhejiang Chinese Medical University, Hangzhou, Zhejiang 310053 China; 2 Key Laboratory of Integrated Oncology and Intelligent Medicine of Zhejiang Province, Affiliated Hangzhou First People’s Hospital, Westlake University, Hangzhou, Zhejiang 310006 China; 3 Department of General Surgery, Affiliated Hangzhou First People’s Hospital, School of Medicine, Westlake University, Hangzhou, Zhejiang 310006 China

**Keywords:** Gastrointestinal cancer, Mendelian randomization, Innate/adaptive immunity, Colocalization, Three-step SMR

## Abstract

**Background:**

Innate/adaptive immunity is the key to anti-tumor therapy. However, its causal relationship to Gastrointestinal (GI) cancer remains unclear.

**Methods:**

Immunity genes were extracted from the MSigDB database. The Genome-wide association studies (GWAS) summary data of GI cancer were integrated with expression quantitative trait loci (eQTL) and DNA methylation quantitative trait loci (mQTL) associated with genes. Summary-data-based Mendelian randomization (SMR) and co-localization analysis were used to reveal causal relationships between genes and GI cancer. Two-sample MR analysis was used for sensitivity analysis. Single cell analysis clarified the enrichment of genes.

**Results:**

Three-step SMR analysis showed that a putative mechanism, cg17294865 CpG site regulating HLA-DRA expression was negatively associated with gastric cancer risk. HLA-DRA was significantly differentially expressed in monocyte/macrophage and myeloid cells in gastric cancer.

**Conclusion:**

This study provides evidence that upregulating the expression level of HLA-DRA can reduce the risk of gastric cancer.

**Supplementary Information:**

The online version contains supplementary material available at 10.1186/s12876-024-03284-x.

## Introduction

GI cancer accounts for 26% of global cancer incidence and 35% of all cancer-related deaths [1]. So far, GI cancer still contributes to a large global cancer burden, with the highest in East Asia [2]. Worryingly, the incidence of GI cancer has shown a gradual upward trend in recent years [3]. Genetic variation, viral infection, obesity, environmental factors, and other interactions may be the basis of disease occurrence [4]. Revealing the complexity behind it may provide new insights into the pathogenesis of GI cancer.

Adaptive immunity, also known as specific immunity, kills tumor cells by mediating specific T cells. Innate immunity is the first line of defense against cancer and plays a key role in coordinating the anti-tumor immune response [5]. In the development of GI cancer, the changes of various components of the tumor microenvironment are the key factors affecting prognosis. Therapeutic strategies based on adaptive immunity, such as Immune-checkpoint blockade (ICB) therapy [6], chimaeric antigen receptors (CARs) T therapy [7], and the Bispecific T cell engager (BiTE) therapy [8], have achieved significant success, although only in a small segment of the audience. Similarly, tumor therapy using innate immunity offers potential treatment options [9]. Studies have shown that knocking down the STING pathway can promote polarization of tumor-associated macrophages in innate immunity and induce apoptosis of gastric cancer cells [10]. Therefore, studying the underlying disease mechanisms of innate or adaptive immunity genes may help identify potential pathogenic factors and therapeutic targets for GI cancer. Although a growing number of studies have shown the presence of innate immunity and adaptive immunity genes in gastrointestinal tumors [11–13], no studies have comprehensively and systematically determined their potential causal relationship with the disease.

Mendelian randomization (MR) is an epidemiological investigation method that uses genetic variants as instrumental variables to measure possible causal relationships between exposure factors and outcome factors [14]. In MR, random assignment of alleles avoids bias from unobserved confounding factors, including environmental factors and lifestyle habits. Genome-wide association studies (GWAS) reveal genetic associations between traits through Single nucleotide polymorphisms (SNPs) and can be combined with gene expression and methylation analysis [15].

Summary-data-based Mendelian randomization (SMR) expands and enriches MR [16]. It combines GWAS summary statistics with QTL data for aggregate integration analysis, prioritizing possible causal relationships. Previous studies have shown that SMR analysis can accurately identify and validate the pathogenic genes of Crohn’s disease [17]. SMR analysis has also demonstrated powerful analytical capabilities in the identification of biomarkers and drug targets in colorectal cancer [18].

In this study, SMR analysis was used to explore the causal relationship between innate/adaptive immune genes and gastrointestinal cancer from a genetic and single-cell perspective. Considering the lack of reliability of SMR alone for identifying cancer pathogenic proteins, colocalization, MR, and heterogeneity of dependent instruments (HEIDI) tests were subsequently performed for sensitivity and heterogeneity analysis. In addition, we used single-cell expression analysis to detect cell type enrichment of the above target genes, thereby demonstrating the reliability of our results from different perspectives.

## Methods

### Study design

**Fig. 1:**
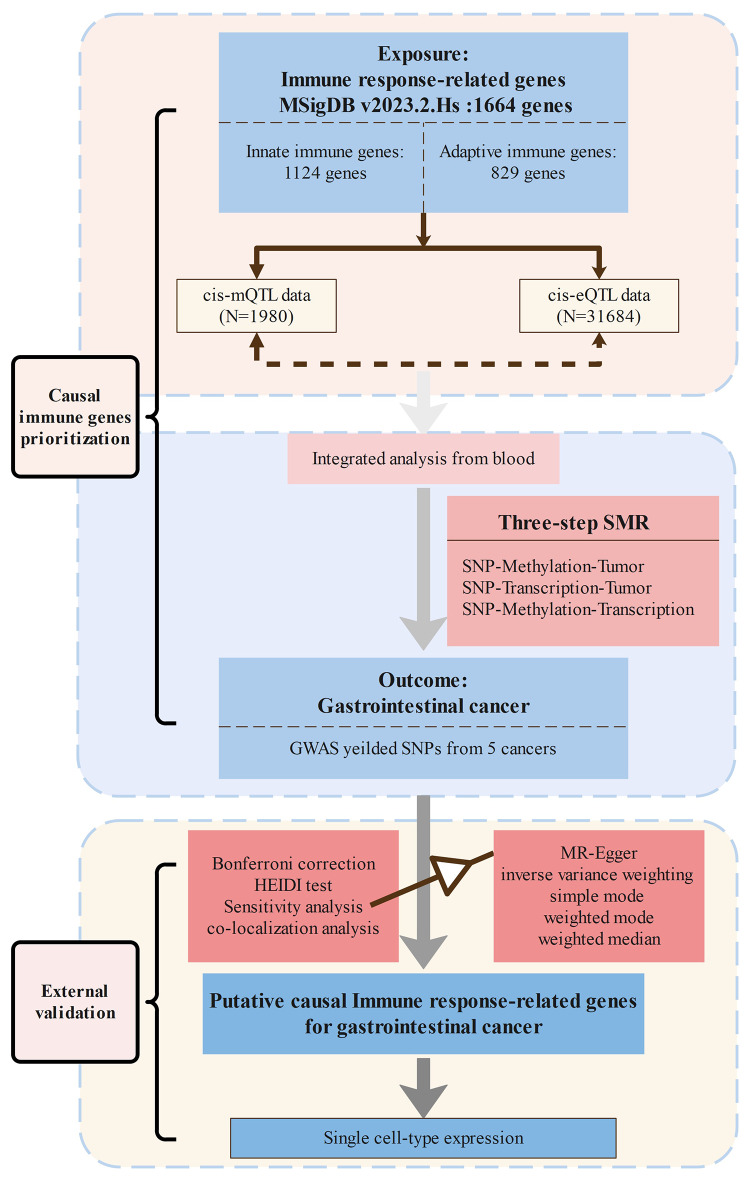
The overall design of this study

Our research workflow is shown in Fig. 1. In brief, we extracted eQTL and mQTL summary data for innate immunity and adaptive immunity genes, integrated them with GWAS summary data for 5 GI cancer, and analyzed the causal relationships between potential genes and traits using three-step SMR, and HEIDI tests. 5 two-sample MR analyses, Bayesian colocalization analysis, statistical power calculations, and phenotypic scanning were used to validate the robustness of causal relationships. In addition, single cell type expression analysis detected cell type-specific expression of target genes on target tumors.

### Data source

To determine potential causal relationships between innate and adaptive immune responses and gastrointestinal tumors, we extracted a list of innate immunity genes and adaptive immunity genes from the MSigDB database (https://www.gsea-msigdb.org/gsea/msigdb/index.jsp). The eQTL summary data obtained by using the eQTLGen consortium contain summary statistical genetic data on blood gene expression from 31,684 individuals [19]. The mQTL summary data were extracted from pooled data from two cohorts (*n* = 1980) [20]. Current studies focused only on cis-eQTL and cis-mQTL. 5 GI cancer (gastric cancer, colon cancer, liver cancer, esophagus cancer, and pancreatic cancer) GWAS summary data were obtained from publicly available databases and included in this study [21–23]. The basic information of QTL and GWAS summary data in this study is shown in Table S1.

### Statistical analysis

The primary analysis consisted of three stages: three-step SMR, sensitivity analysis, and co-localization analysis.

To detect pleiotropic associations between gene expression levels and complex traits, we used the SMR software tool based on the SMR & HEIDI methods [20]. We performed three time SMR analyses in sequence, using DNA methylation sites, gene expression, and GI cancer phenotype as exposure factors or outcomes, and SNPs as genetic tool variables. Step 3 included only the significant signals from steps 1 and 2. In addition, we had strict screening criteria for significant signals, the criteria were as follows: (1). FDR < 0.05 in all three-step SMR; (2). The P value of genome-wide significance < 5 × 10^− 5^ in all eQTL, mQTL, and GWAS; (3). The P value of HEIDI test > 0.05 in all three-step SMR. Simultaneously, the R^2^ and F-statistic were used to estimate the strength of genetic instruments [24]. When the F-statistic was greater than 10, the genetic variant used was considered a strong instrumental variable.

To clarify the stability of the three-step SMR results, we used 5 two-sample MR analysis methods for sensitivity analysis, including MR-Egger, inverse variance weighting (IVW), simple mode and weighted mode, weighted median [25]. In MR-Egger and IVW methods, heterogeneity of individual causal effects was tested by calculating Cochran’s Q statistic, where the P-value of Cochran’s Q test < 0.05 indicated the existence of heterogeneity [26]. The intercept of MR-Egger can be used to indicate whether directional horizontal multidirectivity drove the results of the MR analysis. If the intercept was close to 0, there was no directional multidirectivity, where the P value > 0.05. Wald ratio method was applicable to any proteins with only one instrument.

To refine the results of HEIDI, we performed another Bayesian test on the preliminary results above using the “coloc” R package (https://chr1swallace.github) to estimate the posterior probabilities of shared variables [27]. For the top SNP in the investigated cancer GWAS database, we extracted all SNPs within 100 kb upstream and downstream of the top SNP of the probe in co-localization analysis. P1 (the prior probability of SNP association with GWAS) and P2 (the prior probability of SNP association with QTL) were determined to be 1e-04, P12 = 5e-05 (the prior probability of SNP association with GWAS and QTL). The rest used default parameters to perform co-localization analysis. And, the posterior probability PPH4 > 0.80 was taken as strong evidence.

### Phenotypic scan

PhenoScanner database (http://www.phenoscanner.medschl.cam.ac.uk/) was used to identify relationships between identified genetic variants and other traits [28].

### Single cell type expression analysis

To further assess the cell-type specific expression of target genes on GI cancer. A single cell dataset (GSE183904) was searched in the GEO database (https://www.ncbi.nlm.nih.gov/geo/query/acc.cgi?acc=GSE183904) [29], and the scrna sequencing data of 12 groups of gastric cancer tissues (GSM5573467, GSM5573470, GSM5573472, GSM5573475, GSM5573477, GSM5573478, GSM5573480, GSM5573487, GSM5573489, GSM5573491, GSM5573497, GSM5573501) were integrated and processed by the “Seurat” R package [30]. All cells were removed that had over 3000 expressed genes, or over 15% of UMIs derived from the mitochondrial genome. NormalizeData, ScaleData, and RunHarmony functions were used to Standardization, normalization, and remove batch effects. Then, the cells were clustered using the FindNeighbors and FindClusters functions (Resolution = 0.9) to obtain 22 cell subgroups, and the cells were annotated according to known marker genes. Finally, Clusters were visualized using T-distributed stochastic neighbor embedding (T-SNE) as implemented in Seurat. The FindAllMarkers function was used to compare gene expression levels in cell populations by performing difference analysis on each cell population by the Wilcox rank sum test. We defined an average Log2 fold change (Log2FC) more than 0.5 and a false discovery rate (FDR) adjusted P value less than 0.05 as the enriched gene in each cell type.

## Results

### Integration of GWAS and Immune response-related mQTL/eQTL data from the blood

To determine the role of immune response-related genes in GI cancer (gastric cancer, colon cancer, liver cancer, esophagus cancer, and pancreatic cancer) and explore possible epigenetic mechanisms of gene regulation. We collected 1124 genes associated with innate immunity and 829 genes associated with adaptive immunity, for a total of 1660 genes included as candidates. SNP and DNAm sites of genes associated with innate/adaptive immunity were extracted from blood m/eQTL data using the SMR test. A total of 5654 CpG sites were obtained from innate/adaptive immunity genes and 390,899 SNPs were associated. The mQTL for innate/adaptive immunity genes was then integrated with GWAS data for individual GI cancer. In summary, mQTL and gastric cancer GWAS summary data identified 16 CpG sites, a total of 10 genes (SMR FDR < 0.05 and HEIDI *P* > 0.05) (Table S2).

We also integrated 1791 eQTL probes, which were associated with 8,932,943 SNPs, with GWAS data for GI cancer. We found that 2 genes, HLA-DRA (Betasmr = -0.33) and HLA-DPB1 (Betasmr = -0.18), were negatively associated with gastric cancer (Table S3). Additionally, METTL7A (Betasmr = -0.20) exhibited a protective effect against colon cancer in particular (Table S3).

### Blood methylation regulates gene expression to speculate on putative pathogenic proteins

**Fig. 2:**
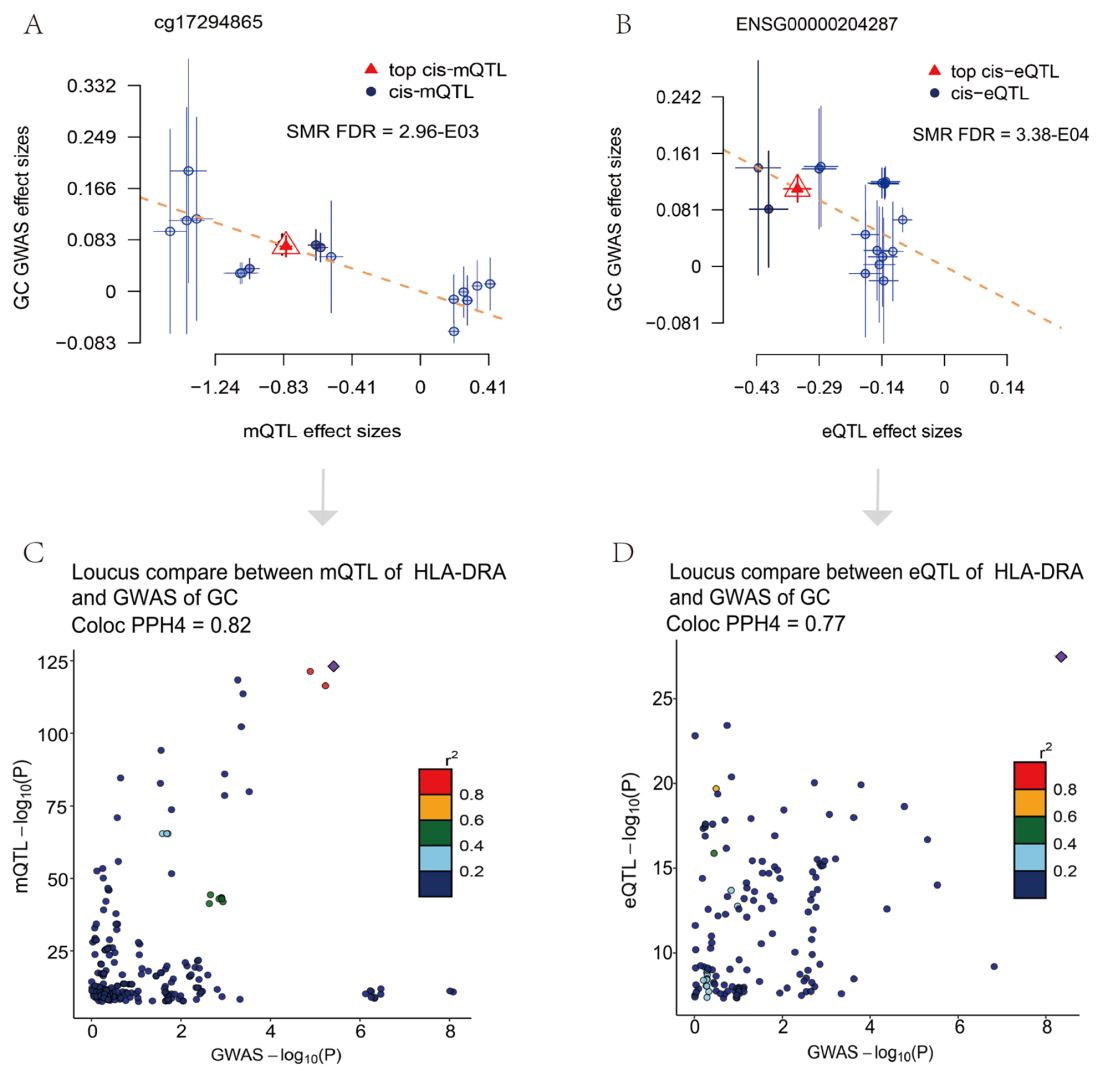
Three-step SMR and co-localization prioritized Potential interactions between immune-related genes and GI cancer. **(A)**. SMR between cg17294865 and gastric cancer GWAS (SMR FDR < 0.05, HEIDI test *P* > 0.05). **(B)**. SMR between ENSG0000204287 and gastric cancer GWAS (SMR FDR < 0.05, HEIDI test *P* > 0.05). **(C)** Locus comparison between cis-mQTL of HLA-DRA and gastric cancer GWAS by co-localization analysis. **(D)**. Locus comparison between cis-eQTL of HLA-DRA and gastric cancer GWAS by co-localization analysis. The r^2^ value indicates the linkage disequilibrium (LD) between the variants and the top SNPs.

**Fig. 3:**
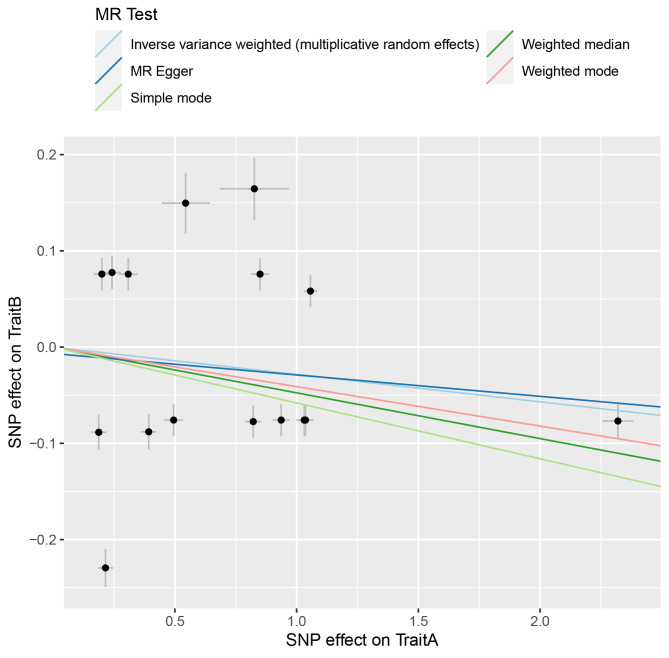
Scatterplot of genetic association between DNA methylation and GWAS of gastric cancer

We hypothesized that SNPs could alter DNAm levels to affect gene expression, thereby explaining plausible causal relationships for the disease. After screening with SMR FDR < 0.05 and HEIDI *P* > 0.05, we found that only HLA-DRA was regulated by a significantly associated methylation site cg17294865 in gastric cancer (Table S4). In colon cancer, although we identified one gene (METTL7A) in our integrated analysis of GWAS summary data and eQTL data, no methylation sites passed the SMR analysis. HLA-DRA, a well-known adaptive immune response gene whose expression is influenced by extracellular antigens and involved in the development of immune responses, was prioritized according to the three-step SMR analysis. DNAm probe cg17294865 was located in the CpG island shores region 640kbp downstream of HLA-DRA. Our study showed that methylation levels at this locus were causally negatively correlated with HLA-DRA (Betasmr= -0.04) expression. Higher methylation levels (Betasmr = -0.09) and elevated levels of HLA-DRA expression (Betasmr = -0.33) may reduce the risk of gastric cancer (Fig. 2A-B). Therefore, it was speculated that a putative mechanism may be that genetic variation up-regulates HLA-DRA expression levels by affecting CpG island shore status, which in turn reduces the risk of gastric cancer. The sensitivity analysis was performed using the two-sample MR method to verify the stability of the SMR analysis results (Fig. 3). As expected, the Two-sample MR results also support the SMR analysis results (Table S5-S7). Our calculated F-statistic results show that each SNP behaves as a powerful exposure tool (Table S1-S3). Bayesian co-localization analysis was used to eliminate confounding due to linkage disequilibrium (LD). Our results show that methylation locus cg17294865 (PPH4 = 0.82) and gene HLA-DRA (PPH4 = 0.77) share a genetic figure with gastric cancer (Fig. 2C-D).

### Phenome-wide scan of identified genetic variants

To further clarify the robustness of the results, the PhenoScanner database was used to rule out possible pleiotropy of the tumors. Our study showed that DNA methylation sites were not found to be associated with all available secondary traits. It was worth mentioning that the causal relationship between colon cancer and its DNA methylation sites was also strong (Table S8).

### Cell type-specific expression in gastric cancer

**Fig. 4:**
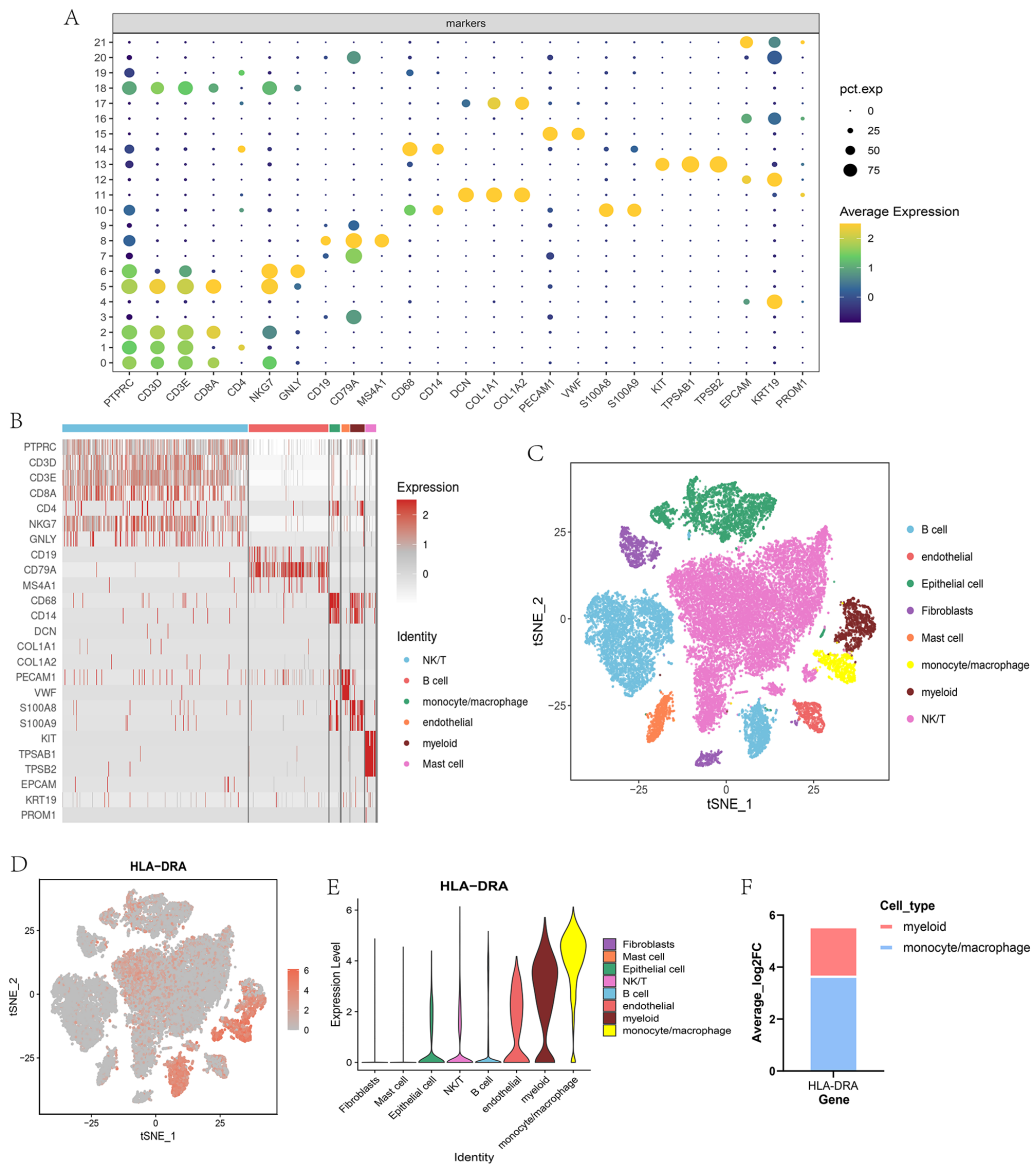
Single cell type expression of HLA-DRA in gastric cancer. **(A)**. Bubble plot showing expression of cell surface markers in 22 clusters. **(B)**. Heat map showing the expression profiles of cell surface markers of each cell type. **(C)**. T-SNE plot showing the distribution of major cell types in gastric cancer tissues. Different colors represent different cell types. (**D-E**). T-SNE plot **(D)** and violin diagram **(E)** showing HLA-DRA gene expression in major cell types of gastric cancer tissue. **(F)**. Histogram showing the enrichment of the HLA-DRA gene in gastric cancer tissues (average Log2FC > 0.5 and FDR < 0.05).

To investigate whether there was a cell type-specific enrichment of HLA-DRA, an adaptive immune-related coding gene, we performed cellular expression analysis by analyzing single-cell RNA-seq data of gastric cancer from the GEO database. RNA-seq data from 12 cancer tissues were unsupervisedly clustered into 22 components. According to the marker genes of each compartment (Fig. 4A), we identified 8 major cell types: NK/T cells, B cells, Epithelial cells, myeloid cells, Fibroblasts, monocyte/macrophage, Mast cells, and Endothelial cells (Fig. 4B-C). We subsequently analyzed the cell-to-cell differences in these eight cell populations, and our study showed that HLA-DRA was enriched in monocyte/macrophage and myeloid cells (Fig. 4D-F).

## Discussion

To the best of our knowledge, this study is the first to identify immune genes potentially causally associated with GI cancer using a multi-omics combined approach. SMR we use expands and enriches the MR. Based on SMR analysis, we hypothesized that SNPs can alter DNA methylation levels to influence gene expression and thus influence tumor pathogenesis. A more stringent screening criterion and the three-time SMR screening gave our results a strong stability. Subsequently, by integrating GWAS data for GI cancer with e/mQTL from peripheral blood, we identified several possibilities but prioritized an adaptive immune-related gene (HLA-DRA) and a methylation site (cg17294865) in gastric cancer. Previous studies have shown that HLA-DRA was associated with the risk of gastric cancer development [31]. However, the potential mechanism of HLA-DRA in gastric cancer had not been characterized. Our study showed that HLA-DRA was specifically highly expressed in monocytes/macrophages and myeloid cells and reduced the risk of gastric cancer. Our study provided strong evidence for the underlying mechanisms by which genetic variation, methylation, and gene expression of HLA-DRA were associated with the trait of gastric cancer. Similarly, the gene expression of HLA-DPB1 showed a sketchy causal relationship. It is worth mentioning that our study showed a causal relationship between gene expression of METTL7A and colon cancer, despite the absence of methylation sites according to the SMR analysis. Furthermore, we further clarified the differential expression of HLA-DRA in monocytes/macrophages and myeloid cells in gastric cancer through single-cell omics studies.

Innate immunity was the innate cornerstone of the anti-tumor immune response. In recent years, anti-tumor therapies based on innate immunity have demonstrated potent activity. There was still a need to explore more novel immune checkpoints to enrich the understanding of innate immunity’s anti-tumor mechanisms and thereby stimulate the full potential of the human immune system [9]. METTL7A was an RNA N6-methyladenosine (m6A) methyltransferase involved in methylation and lipid metabolism [32], and an innate immunity gene. Studies have shown that METTL7A was significantly under-expressed in colon cancer tumor tissues, and its expression level was predictive of colon cancer with high accuracy [33]. This was consistent with our findings that METTL7A may represent a potential therapy target for colon cancer.

Adaptive immunity was essential for protective immunity against tumors. It mediates cellular and humoral immunity to prevent and limit cancer through immunological detection [34]. HLA-DPB1 (Major Histocompatibility Complex, Class II, DP Beta 1) was a Protein Coding gene. It plays a central role in the adaptive immune system by presenting peptides derived from extracellular proteins. Previous studies have shown that HLA-DPB1 is a susceptibility locus for colon cancer [35]. Moreover, In our study, we found a negative association between HLA-DPB1 and gastric cancer. The mechanism of HLA-DPB1 in gastric cancer remains to be further explored.

HLA-DRA was an HLA class II alpha chain paralogue, an adaptive immunity gene involved in antibody-mediated immune response and macrophage activation, ultimately affecting tumor cell growth [36–38]. Research showed that HLA-DRA can guide ICB in non-small cell lung cancer and ER-negative breast cancer [39, 40]. It was also differently expressed in different types of tumors. HLA-DRA exhibited upregulation in colon cancer and hepatocellular carcinoma [41, 42] while demonstrating downregulation in breast cancer [43]. Our study suggested that elevated levels of HLA-DRA expression and higher methylation levels may reduce the risk of gastric cancer. These findings highlighted that HLA-DRA gene expression was causally associated with gastric cancer and may represent a potential immunity target for gastric cancer therapy.

The strength of this study is that we provide a comprehensive and systematic assessment of the causal relationship between innate/adaptive immunity and GI cancer. At the same time, the inclusion of a larger sample size of different GI cancer GWAS summary data in the study allows us to draw more robust conclusions. The final results are sifted by a three-step SMR and sensitivity analysis is performed by additional MR analysis and co-localization analysis. These also show the robustness of our results. The study included only individuals of European ancestry, thus reducing the bias from different genetic backgrounds. Finally, single cell type expression analysis provides updated insights into the underlying pathogenesis of HLA-DRA in gastric cancer.

There are some limitations to this study. Although we include large sample sizes of GWAS summary data, protein QTL (pQTL) summary dates for immune-related genes are lacking. In addition, among the eQTL and mQTL included in the study, there is no information on genetic variation on the X and Y chromosomes. Second, the posterior probability PPH4 of co-localization of the HLA-DRA gene with gastric cancer GWAS in our study was 0.77, but PPH4 ≥ 0.80 was considered strong evidence of Bayesian co-localization. Despite previous findings, many sites with PPH4 ≥ 0.5 appear qualitatively consistent with the co-localization provided by PPH4 ≥ 0.8 [44]. Third, our study only includes eQTL and mQTL in the cis-region. Trans domains may also affect disease regulatory networks, but it is difficult to explain their causal relationship to disease [19]. Fourth, although our study included single-cell analysis, additional genomic data at different molecular levels are needed to further explore the pathogenesis.

## Conclusion

Through Mendelian randomization analysis and single-cell analysis, our results suggest a potential pathogenic mechanism that the expression level of HLA-DRA, which is mainly expressed in monocytes/macrophages and myeloid cells, is inversely correlated with the risk of gastric cancer.

### Electronic supplementary material

Below is the link to the electronic supplementary material.

Supplementary Material 1: Table S1. Information of e/mQTL and GWAS datasets used in the current study. Table S2. SMR analysis from eQTL to tumor (FDR < 0.05 and p_HEIDI > 0.05). Table S3. SMR analysis from mQTL to tumor (FDR < 0.05 and p_HEIDI > 0.05). Table S4. SMR analysis from mQTL to eQTL (FDR < 0.05 and p_HEIDI > 0.05). Table S5. Sensitivity analysis used the TwoSampleMR package on the association between gene expression to GWAS. Table S6. Sensitivity analysis used the TwoSampleMR package on the association between DNA methylation to GWAS. Table S7. Sensitivity analysis used the TwoSampleMR package on the association between DNA methylation to gene expression. Table S8. Phenome-wide scan of the association between identified SNPs with other disease traits using the PhenoScanner database.

## Data Availability

The data used in this study are available from the published articles and are listed in Table S1. Single-cell RNA-seq data for human gastric cancer tumor tissue are available in the GEO database (www.ncbi.nlm.nih.gov/geo/query/acc.cgi? acc=GSE183904).

## Abbreviations

GI: Gastrointestinal
ICB: Immune-checkpoint blockade
CARs: Chimaeric antigen receptors
BiTE: Bispecific T cell engager
MR: Mendelian randomization
GWAS: Genome-wide association studies
SNPs: Single nucleotide polymorphisms
SMR: Summary-data-based Mendelian randomization
eQTL: expression quantitative trait locus
mQTL: DNA methylation quantitative trait locus
pQTL: protein abundance quantitative trait locus
HEIDI: The heterogeneity in dependent instruments
GEO: Gene Expression Omnibus
T-SNE: T-distributed stochastic neighbor embedding
FDR: False discovery rate
LD: Linkage disequilibrium
RNA-seq: RNA sequencing
